# Structural Aspects of Arylpiperazines as Aminergic GPCR Ligands

**DOI:** 10.3390/molecules30122545

**Published:** 2025-06-11

**Authors:** Agata Bartyzel, Beata Cristóvão, Agnieszka A. Kaczor

**Affiliations:** 1 Department of General and Coordination Chemistry and Crystallography, Institute of Chemical Sciences, Faculty of Chemistry, Maria Curie-Sklodowska University in Lublin, Maria Curie-Sklodowska Sq. 2, 20-031 Lublin, Poland; agata.bartyzel@mail.umcs.pl (A.B.); beata.cristovao@mail.umcs.pl (B.C.); 2 Department of Synthesis and Chemical Technology of Pharmaceutical Substances with Computer Modeling Laboratory, Faculty of Pharmacy, Medical University of Lublin, 4A Chodźki St., 20-093 Lublin, Poland; 3 School of Pharmacy, University of Eastern Finland, Yliopistonranta 1, P.O. Box 1627, 70211 Kuopio, Finland

**Keywords:** arylpiperazines, CNS disorders, GPCRs, molecular modeling, pharmacophore models, structure–activity relationship, X-ray structure

## Abstract

Arylpiperazines are considered a “privileged scaffold” in medical chemistry due to their versatility and modular structure, enabling modifications towards diverse molecular targets with desired potency, selectivity, and pharmacokinetic properties. In particular, arylpiperazines are aminergic G protein-coupled receptor (GPCR) ligands and neurotransmitter transporter inhibitors, making this group of compounds attractive in central nervous system (CNS) drug discovery for treating schizophrenia, depression, sleep disorders, and Parkinson’s disease (PD). Furthermore, arylpiperazines may possess anticancer properties and can modulate some molecular targets involved in this disease. This review focuses on the structural aspects of arylpiperazines as aminergic GPCR ligands. The review centers on biologically active arylpiperazines with known X-ray structures, providing a detailed discussion of the conformations in the solid state. Next, their interactions with the aminergic GPCRs, based on experimental and molecular modelling studies, are addressed, making this review a comprehensive resource for medicinal and structural chemists working on arylpiperazines.

## 1. Introduction

The concept of “privileged structures” in medicinal chemistry was first used by Evans in 1988 [1,2], characterizing them as simple structural subunits occurring in the molecules of numerous drugs, with differentiated therapeutic uses and affinities to various receptors [3]. This term later evolved into terms such as “molecular framework”, “chemotype”, “molecular fragment”, “molecular scaffold”, and “privileged scaffold”, meaning that some scaffolds may have privileged characteristics, being recognized molecularly by distinctive receptors [3,4].

Arylpiperazines can be considered a “privileged scaffold”, particularly for the central nervous system (CNS) [5] and anticancer [6] drugs. Arylpiperazines are versatile and modular, facilitating modifications to obtain compounds with desired affinity, selectivity, and pharmacokinetic properties. As CNS active compounds, arylpiperazines are mainly aminergic G protein-coupled receptor (GPCR) ligands or neurotransmitter transporter inhibitors. Regarding GPCRs, they target serotonin [7,8], dopamine [9], and adrenergic [10] receptors and are developed for treating schizophrenia, depression, anxiety, sleep disorders, and Parkinson’s disease (PD). *N*-arylpiperazines can also have antihistamine, anti-inflammatory, and antihypertensive activities [6].

**Figure 1:**
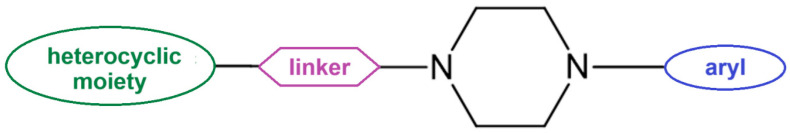
General structure of *N*-arylpiperazine derivatives.

The general structure of arylpiperazines involves the central piperazine ring, an *N*-aryl group, and a linker connecting another moiety, often a heterocyclic group with another nitrogen atom (see Figure 1). In particular, the long-chain arylpiperazine scaffold is a versatile template for designing CNS drugs that target serotonin [11] and dopamine receptors [12].

Since 2007, the structural biology of GPCRs has undergone a transformative shift with the advent of high-resolution crystallography and later cryo-electron microscopy (cryo-EM). The first high-resolution structure of a non-visual GPCR, the β_2_-adrenergic receptor, crystallized using lipidic cubic phase and T4 lysozyme fusion, revealed crucial insights into ligand binding and receptor stabilization. Subsequent structures across classes A, B, C, and F illuminated conserved activation mechanisms such as the outward movement of transmembrane helix 6 (TM6), intracellular cavity formation, and G protein coupling. Cryo-EM further enabled the visualization of full receptor–G protein complexes and dynamic conformational states, expanding our understanding of allosteric modulation, biased agonism, and receptor oligomerization. Together, these advances have reshaped drug discovery by mapping structural determinants of specificity, efficacy, and signaling bias in GPCR-targeting compounds.

This review focuses on the structural aspects of arylpiperazines as aminergic GPCR ligands. Arylpiperazines with known molecular structures are described, with particular attention given to their conformations in the solid state. This is complemented by the discussion of their interactions with aminergic GPCRs based on experimental studies or molecular modeling. The structure–activity relationships of arylpiperazines can be found in earlier reviews [5,7,11]. It should be stressed that to our best knowledge, this review is the first comprehensive resource focusing on the structure of arylpiperazines in the context of their biological activity. Some earlier reviews focus on the exploration of the binding of arylpiperazines to the D_2_ receptor [9] or molecular docking models of arylpiperazines to the 5-HT_1A_, 5-HT_2A_, and 5-HT_7_ receptors [13]. Our review is of interest to medicinal and structural chemists working on arylpiperazines.

## 2. Solid-State and Bioactive Conformations of Arylpiperazines

The arrangement of atoms in a solid state is governed by the intermolecular interactions and the specific packing arrangement of the molecules within the crystal lattice, which can be determined using techniques such as X-ray diffraction. The conformation in the solid state can be significantly different from the conformation of the same molecule in a solution in which molecules are free to move and can adopt various conformations, often determined by solvent interactions and temperature. The unknown effect of a receptor’s environment on a ligand’s conformation presents a major challenge in predicting feasible bioactive conformations, particularly if the receptor is not well defined [14]. The problem of a drug’s bioactive conformation can be addressed using molecular modeling techniques, such as molecular docking and molecular dynamics, or experimental techniques, including X-ray crystallography or cryo-electron microscopy (cryo-EM) of ligand–receptor complexes, all facilitating structure-based drug design [15], in particular, in the field of GPCRs [16,17].

### 2.1. Aripiprazole

**Figure 2:**
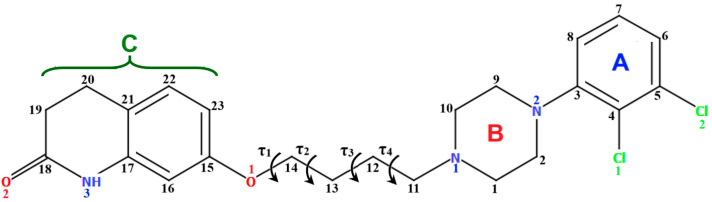
Scheme of the molecular structure of aripiprazole.

Aripiprazole (Figure 2), also known by the brand name Abilify, is one of the most popular antipsychotic drugs. It is an arylpiperazine derivative consisting of four units: a dichlorophenyl ring, a piperazinyl moiety, an *n*-butoxyl linker, and a dihydrocarbostyril fragment.

**Figure 3:**
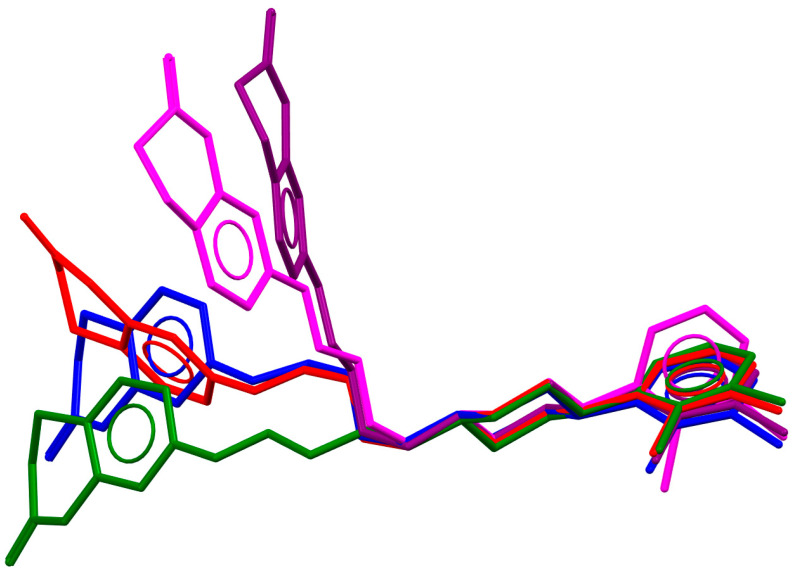
An overlay of aripiprazole molecular conformation from five polymorphs (red—MELFIT01, green—MELFIT02, purple—MELFIT07, blue—MELFIT09, magenta—MELFIT19) [18].

Many polymorphic structures of this compound (Table 1), as well as its solvates, salts, and co-crystals, can be found in the CSD database [18]. The piperazine ring adopts a chair conformation in all polymorphic structures (MELFIT, MELFIT01, MELFIT06, MELFIT07, MELFIT08, MELFIT19, and MELFIT20) [19]. In polymorphs of aripiprazole, the phenyl ring is inclined to the piperazine ring by 35.23–48.18° (Table 1). The −(CH_2_)_4_− linker (C11−C12−C13−C14) generally has an antiperiplanar conformation, and the torsion angle *τ_3_* is ~180° (Table 1). The polymorphic compound METFIT07 is an exception, in which the angle *τ_3_* is 58.3°, indicating a *gauche* conformation. The torsion angles *τ_2_* (O1−C14−C13−C12) and *τ_4_* (C13−C12−C11−N1) have a major influence on the structure of the molecule, i.e., whether it is extended or bent (Figure 3). It may be seen that the *n*-butoxyl fragment in these polymorphs adopts three different conformations (Table 1), namely *anti–anti* (MELFIT, MELFIT02, MELFIT03, MELFIT04, MELFIT05, MELFIT06, MELFIT08, MELFIT15, MELFIT16, MELFIT17, and MELFIT20), *gauche–gauche* (MELFIT01, MELFIT09, MELFIT10, MELFIT11, MELFIT12, MELFIT13, MELFIT14, and MELFIT18), and *anti–gauche* (MELFIT07 and MELFIT19).

Aripiprazole, as a third-generation antipsychotic (TGA), has an affinity to several GPCRs (see Table 2) [27]. In particular, it is a ligand of the dopamine D_2_ receptor with *K_i_* values of 0.74 nM and 3.3 nM to D_2L_ and D_2_ receptors, respectively. It is a partial or biased agonist of the dopamine D_2_ receptor, sometimes termed the dopamine stabilizer [28,29,30]. It also displays affinity to several serotonin receptors (see Table 2). It acts as a partial agonist at the serotonin 5-HT_1A_ receptors and an antagonist or weak partial agonist at the serotonin 5-HT_2A_ receptors.

There are several reports regarding aripiprazole interactions with aminergic GPCRs at the molecular level. An early study by Salmas et al. [31] reported a molecular docking pose of aripiprazole in the dopamine D_2_ receptor modeled on the β_2_ adrenergic receptor template. It was followed by a quantum chemical estimation of the binding affinity. Aripiprazole exhibited an extended conformation in the receptor binding pocket. Asp114^3.32^ strongly interacts with this ligand in the 7TM domain, as expected [31].

Subsequent chemoinformatics and molecular docking studies were employed to investigate 225 complexes of 75 schizophrenia antipsychotics, including aripiprazole, with the dopamine receptor subtypes D_2_, D_3_, and D_4_ [32]. It was found that aripiprazole and other atypical antipsychotics have stronger interactions with the D_2_ and D_4_ receptors, but their interactions with the D_3_ receptor are slightly weaker, which resembles the behavior of dopamine. Recently, aripiprazole was investigated as a drug to alleviate the high prolactin levels induced by amisulpride via distinct molecular mechanisms using network pharmacology and molecular docking [33]. The core identified targets of aripiprazole include MAPK3 (mitogen-activated protein kinase 3), PPARG (peroxisome proliferator-activated receptor gamma), the D_2_ receptor, and ESR1 (estrogen receptor 1), providing new insights into the mechanisms of this drug in schizophrenia treatment.

**Figure 4:**
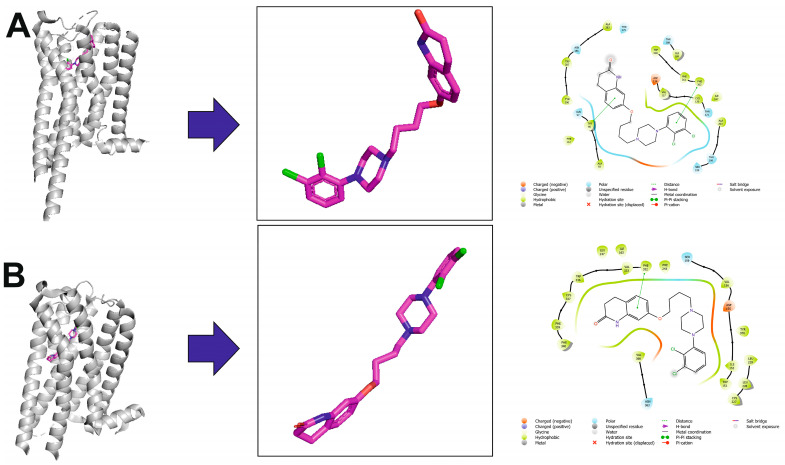
Aripiprazole in complex with the serotonin 5-HT_1A_ (**A**) and 5-HT_2A_ (**B**) receptors. The left part of the panels shows ligand–receptor complexes. Protein is shown as a grey cartoon representation. Ligands are shown as sticks with magenta carbon atoms. The middle panels show the aripiprazole bioactive conformation, following the ligand orientation in the binding pocket. Aripiprazole is depicted as sticks with magenta carbon atoms. Hydrogen atoms are omitted for clarity. The right panels show 2D interaction maps generated with Schrödinger Maestro.

The molecular interactions of aripiprazole with serotonin receptors can be illustrated based on two ligand–receptor complexes available in Protein Data Bank, i.e., the 5-HT_1A_ receptor in an active conformation in complex with G_i_ (PDB ID: 7EZ2, cryo-EM structure) [34] (see Figure 4A) and the 5-HT_2A_ receptor in an inactive conformation (PDB ID: 7VOE, X-ray structure) [35] (see Figure 4B). In the case of the complex of aripiprazole with the 5-HT_1A_ receptor, the complexes with closely related 5-HT_1B_, 5-HT_1D_, and 5-HT_1E_ receptors were obtained using molecular modeling. Structural analysis of these complexes revealed the basis of the molecular recognition of aripiprazole. In the 5-HT_1A_ receptor, the extracellular end of transmembrane helix 7 (TM7), which contributes directly to forming the extended ligand-binding pocket, is displaced outward by roughly 3 Å compared to its position in the 5-HT_1B_, 5-HT_1D_, and 5-HT_1E_ receptors. Along with residues Phe112^3.28^ and Tyr96^2.63^, TM7 helps stabilize the quinolinone moiety of aripiprazole in 5-HT_1A_. In contrast, in the 5-HT_1B_, 5-HT_1D_, and 5-HT_1E_ receptors, TM7 is positioned more inwardly, and the presence of a bulkier Trp^3.28^ residue (equivalent to Phe122^3.28^ in 5-HT_1A_), which would overlap with the same binding space of the quinolinone group, leads to a reduced aripiprazole affinity. Importantly, a prominently positioned cholesterol molecule is found lodged between TM1 and TM7 in 5-HT_1A_, acting as a structural chaperone for these two helices. This cholesterol assists in shaping the ligand-binding pocket and helps maintain TM1 and TM7 in a configuration that favors the binding of aripiprazole, thereby enhancing its affinity. This finding supports the known regulatory role of cholesterol in 5-HT_1A_ function. In contrast, no cholesterol is detected at the equivalent location in 5-HT_1B_, 5-HT_1D_, or 5-HT_1E_ structures [34]. Aripiprazole as a TGA also acts as an antagonist or weak partial agonist to the 5-HT_2A_ receptor. In the crystal structure of the ligand–receptor complex, it adopts an unexpected ‘upside-down’ pose in the 5-HT_2A_ receptor binding pocket, with secondary pharmacophores inserted in a similar way to a ‘bolt’ (Figure 4B) [35].

In general, arylpiperazines can be designed as bitopic ligands that interact with the primary binding pocket (PBP) and the secondary binding pocket (SBP), also called the extended or allosteric pocket. The SPB is situated in the extracellular vestibule and is less conserved than the main binding pocket; thus, it can modulate the affinity, selectivity, and functional activity of the ligands. The example of aripiprazole shows that the same ligand may adopt a completely different pose in the subtypes of the same receptors. While Asp^3.32^ remains the main anchoring point for the piperazine moiety, any further generalizations of the binding mode should be made with care. One can identify, however, key residues involved in the SBP, which include residues in the extracellular ends of transmembrane helices TM1, TM2, TM7 or TM3, TM5, and extracellular loop ECL2. For example, in the aripiprazole-5-HT_1A_ receptor complex, these residues involve Tyr^2.63^, Gln^2.64^, and Tyr^7.42^, while in the aripiprazole-5-HT_2A_ receptor complex, they involve Trp^3.28^ and Ile^3.29^.

### 2.2. N-Arylpiperazines

#### 2.2.1. General Overview

*N*-arylpiperazines, including long-chain arylpiperazines (LCAPs), are a structurally diverse group of compounds. The central piperazine ring is connected with an aryl or hetrocyclic group and on the other side with a so-called terminal group via a linker [11]. The classification and a wide variety of examples of this group of compounds can be found elsewhere [11]. Attention is focused on compounds that conform to the pattern presented in Figure 1.

The CSD database [18] also contains other arylpiperazine derivatives that have been investigated for the treatment of psychiatric conditions (Table 3). Their general structure is shown in Figure 1. Similarly to the aripiprazole, the piperazine ring in all derivatives has a chair conformation. In the structure, the linker may be a flexible chain (aliphatic –(CH_2_)_n_– chain or aliphatic chain containing a heteroatom) or may have a conformation restricted by the inclusion of constrained moieties such as rings or double bonds [36,37,38]. In addition to the arylpiperazines shown in Figure 1, compounds without a linker and/or heterocyclic moiety are attracting attention. Examples of the compounds being investigated as antipsychotics are BAHRIO, BAHROU, POVSAX, PUDGOK, QENURA, and QENSOV (Table 3).

#### 2.2.2. Arylpiperazine Derivatives Containing −(CH_2_)_n_− Spacer

**Figure 5:**
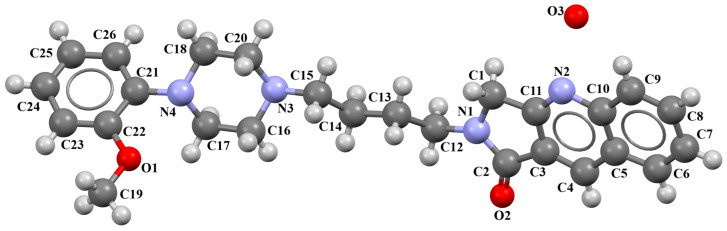
The molecular structure of BIJBON [18,19,40].

Cappelli and co-workers synthesized a hydrated arylpiperazine derivative, BIJBON (displaying a high affinity to the 5-HT_1A_ receptor, *K_i_* of 15 ± 6 nM), in which structure four units can be distinguished: a methoxyphenyl group, a piperazine, a −(CH_2_)_4_− linker, and a fused ring system (Figure 5). The plane of the piperazine ring, formed by non-hydrogen atoms (N4/C17/C16/N3/C20/C18), is inclined to the plane of the phenyl ring (C7/C9/C14/C20/C6/C18) by 47.39° and to the plane of the fused ring system (N1/C1/C11/N2/C10/C9/C8/C7/C6/C5/C4/C3/C2) by only 21.69°. The −(CH_2_)_4_− linker (C12−C13−C14−C15) has an antiperiplanar conformation, and the torsion angle is −173.4(3)°. This spacer links the 2,3-dihydro-1*H*-pyrrolo[3,4-*b*]quinolin-1-one substituent to the piperazinyl moiety in an *anti–anti* conformation with torsion angles of 176.9(3)° (N3−C15−C14−C13) and −178.8(3)° (N1−C12−C13−C14) [40].

Furthermore, they performed molecular docking studies with AutoDock to better understand the ligand-5-HT_1A_ receptor interactions [40]. In the absence of an experimental structure of the receptor, homology modeling with multiple templates was used. The most interesting results were obtained for two potent arylpiperazine ligands, containing a combination of the angularly fused pyrrolidone components with the pentamethylene spacer and the 2-methoxyphenyl arylpiperazine moiety [40]. Both compounds establish a T-shaped interaction with the hydrophobic Phe166 and hydrogen bonds with both Asn217 and Arg244. An additional charge-assisted hydrogen bond was found between the protonated piperazine nitrogen atom and the carboxylic group of Asp78. It should be noted, however, that the numbering of the residues in the reported 5-HT_1A_ receptor model is incorrect. Asp78 is probably Asp1163.32, but the other mentioned residues are difficult to assign using the correct Ballesteros–Weinstein nomenclature.

**Figure 6:**
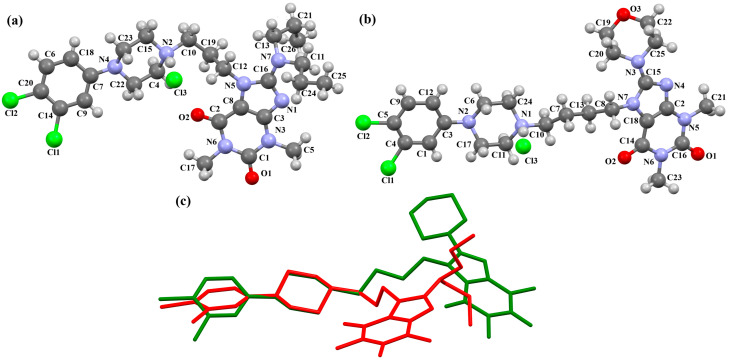
The structure of (**a**) CAHLAB; (**b**) CAHLEF [18,19,41]. The water molecules have been omitted. (**c**) A comparison of the conformations of the two structures of CAHLAB (red) and CAHLEF (green).

The compounds CAHLAB and CAHLEF were synthesized as hydrated arylpiperazine hydrochloride salts with a positive charge on the N15 atom of the piperazine ring (Figure 6). They display an affinity to the serotonin 5-HT_1A_, 5-HT_2A_, 5-HT_6_, 5-HT_7_, and dopamine D_2_ receptors. In CAHLAB, the phenyl ring plane (C7/C9/C14/C20/C6/C18) is significantly rotated concerning the piperazine ring plane (N4/C22/C4/N2/C15/C23). The dihedral angle between the planes formed by the non-hydrogen atoms is 27.26°. In contrast, in the case of CAHLEF, the planes formed by the phenyl (C3/C1/C4/C5/C9/C11) and piperazine (N2/C6/C24/N1/C11/C17) rings are almost parallel, and the dihedral angle is 5.39°. Another significant difference in the structure of the compounds is the length of the chain that connects the pyrazine ring to the heterocyclic ring. In the structure of CAHLAB, the aliphatic linker consists of three methylene groups and adopts a *gauche–anti* conformation with torsion angles of −70.8(2)° for N2−C10−C19−C12 and 178.3(1)° for C10−C19−C12−N2, respectively. The result is the bending of the molecule. In the structure of CAHLEF, the four-member spacer (–(CH_2_)_4_–) connecting two rings adopts an *anti–anti* conformation with torsion angles of −176.5(2)° for N1−C10−C7−C13 and −171.3(2)° for C7−C13−C8−N2, respectively. In addition, the −(CH_2_)_4_− linker (C10–C7–C13–C8) also has an antiperiplanar conformation with a torsion angle of −167.0(2)°. In both structures, the planes of the fused ring system and piperazine ring are rotated by 23.04° for CAHLAB and 20.97° for CAHLEF. A comparison of the conformations of these two structures is shown in Figure 6c.

Molecular docking was performed to rationalize the interactions of selected arylpiperazinylalkyl derivatives of 8-amino-1,3-dimethylpurine-2,6-dione with the receptors at the molecular level. The molecule maintains a consistent shape across the homology models of the 5-HT_1A_ receptor. Its arylpiperazine moiety possesses a protonated nitrogen atom that forms a charge-stabilized hydrogen bond with Asp116^3.32^, while the aryl ring is involved in π–π interactions with Phe362^6.52^ and a cation π interaction with Lys191. Analysis of the binding mode indicates that adding a methoxy group at the 2-position of the aryl ring is the most favorable modification in this series of compounds. This substitution enables hydrogen bonding with Lys191 without causing steric clashes with other residues in the binding site. The 1,3-dimethylpurine-2,6-dione segment contributes to stabilizing the ligand–receptor interaction via π–π stacking with Tyr96^2.64^, and its substituent at the 8-position occupies a non-specific cavity located beneath the second extracellular loop (ECL2). While this part of the molecule does not significantly impact the binding affinity for the 5-HT_1A_ receptor, it may influence activity at other receptor subtypes, such as 5-HT6, as suggested by the observed activity pattern—though more studies are needed to clarify this effect [41]. The compounds in this series exhibit a strong binding affinity for the dopamine D_2_ receptor, primarily due to the incorporation of a well-established arylpiperazine scaffold. Functional assays have shown that several of these compounds act as partial agonists at the D_2_ receptor. Structure–activity relationship (SAR) analysis suggests that the secondary terminal segment of the molecules may play a key role in triggering their intrinsic activity. In the modeled compound, the arylpiperazine moiety interacts with the D_2_ receptor’s Asp^3.32^ and Phe^6.51/6.52^ residues, similar to its interaction in the 5-HT_1A_ receptor, while the 8-piperidine-1,3-dimethylpurine-2,6-dione moiety occupies a less conserved cavity, having aromatic contacts with Tyr^7.35^ and a hydrogen bond with Ser^7.36^ [41]. The proposed binding mode of novel compounds resembles the binding of aripiprazole.

**Figure 7:**
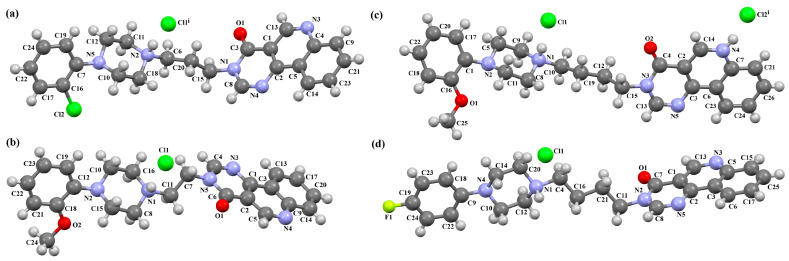
The structure of (**a**) EZEYUE (symmetry code (i) x−1,y,z), (**b**) EZEZAL, (**c**) EZEZEP (symmetry code (i) x,y−1,z), (**d**) EZEZIT [18,19,43]. The solvent molecules have been omitted.

Lewgowd et al. synthesized four arylpiperazine derivatives (EZEYUE, EZEZAL, EZEZEP, and EZEZIT; Figure 7) in the form of the solvated hydrochloride salts as potent 5-HT_1A/2A_ and 5-HT_7_ receptor ligands [43]. The positive charge is localized on the nitrogen atom of the piperazine connected to the aliphatic spacer. Additionally, in the case of EZEZAL, one of the nitrogen atoms of pyrimido[5,4-*c*]quinolin-4(3*H*)-one (N4) also occurs as zwitterion (Figure 7b). Compounds are composed of the same building units, which differ in substituents in the phenyl ring and/or in the length of the linker. In the EZEYUE structure, the substituent in the phenyl ring is a chlorine atom. The plane of the phenyl ring (C7/C16/C17/C22/C24/C19) is rotated by an angle of 53.69° with respect to the plane of the piperazine ring (N5/C12/C11/N2/C18/C10). A slightly smaller rotation is observed for the plane of the fused ring system (N1/C8/N4/C2/C5/C14/C23/C21/C9/C4/N3/C13/C1/C3) and the piperazine ring, for which the dihedral angle is 43.70°. The aliphatic trimethylene chain adopts an *anti–anti* conformation with the torsion angles of N2−C6−C20−C15 and C6−C20−C15−N1 being 168.8(2)° and 164.1(2)°, respectively.

**Figure 8:**
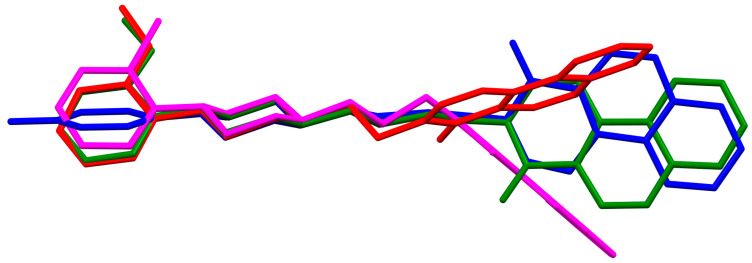
An overlay of molecules (EZEYUE—magenta, EZEZAL—red, EZEZEP—green, EZEZIT—blue) showing the rotation of the phenyl ring and the spacer effect. Hydrogen atoms and solvent molecules have been omitted for clarity [18,19,43].

In the case of EZEZAL, the benzene ring contains a methoxy substituent. The phenyl ring plane (C12/C18/C21/C22/C23/C19) is much less rotated with respect to the piperazine ring, with the dihedral angle of the respective planes formed by the non-hydrogen atoms being 31.94°. However, a much greater rotation is observed in the case of the fused ring system (N5/C4/N3/C1/C3/C13/C17/C20/C14/C9/N4/C5/C2/C6) with respect to piperazine (N2/C10/C16/N1/C8/C15), in which the angle is 77.49°. There is a shorter spacer (−(CH_2_)_2_−) connecting the fused rings to the piperazine ring in an antiperiplanar mode, with the torsion angle of N1−C11−C7−N5 being 179.7(2)°. EZEZEP contains a methoxyphenyl substituent in its structure similar to EZEZAL but has a longer aliphatic linker consisting of four methylene groups. In this compound, the angles between the planes formed by the non-hydrogen atoms of the respective rings are comparable to those of the preceding compound; i.e., the dihedral angles between the plane of the piperazine ring (N2/C5/C9/N1/C8/C11) and the planes of the phenyl ring (C1/C16/C18/C22/C20/C17) or the fused ring system (N3/C13/N5/C3/C6/C23/C24/C26/C21/C7/N4/C14/C2/C4) are 30.04° and 75.30°, respectively. The tetramethylene linker adopts an *anti*, *ant, anti*−conformation with the torsion angles of N1−C10−C19−C12, C10−C19−C12−C15 and C19−C12−C15−N3 being −173.8(1)°, −170.1(1)°, and −166.1(1)°, respectively. The last reported compound, EZEZIT, also contains a four-membered linker, and the phenyl ring is substituted with fluorine in the *para* position. The presence of the substituent in the *para* position, rather than *ortho* as in the other compounds, probably causes the plane of the phenyl ring (C9/C22/C27/C19/C23/C18) to be only slightly rotated by an angle of 3.77° with respect to the plane of the piperazine ring (N4/C10/C12/N1/C20/C14). Additionally, the plane of the fused rings (N2/C8/N5/C2/C3/C6/C17/C25/C15/C5/N3/C13/C1/C7) is almost perpendicular to the plane of the piperazine (89.39°). The −(CH_2_)_4_− linker (C4−C16−C21−C11) has an antiperiplanar conformation, and the torsion angle is −175.5(3)°. This spacer links the pyrimido[5,4-*c*]quinolin-4(3*H*)-one substituent to the piperazinyl moiety in an *anti–anti* conformation with torsion angles of −165.6(3)° (N1−C4−C16−C21) and −169.9(3)° (C16−C21−C11−N2). A comparison of the conformations of the EZEYUE, EZEZAL, EZEZEP, and EZEZIT structures is shown in Figure 8.

Lewgowd et al. [43] also performed extensive molecular modeling studies to address the problem of novel ligands’ conformation. They showed that extended conformations are preferred for solvent simulations, whereas in vacuum, bent geometries dominated. The results of in silico conformation analysis are in agreement with the 2D NMR studies. To complete the research on the conformational preferences of the studied compounds, they performed a flexible docking study using BioSolveIT FlexX 2.0.3 for a population of 100 5-HT_1A_ receptor models. In the best-scoring complexes, the key arylpiperazine moiety was directed deep inside the receptor, between the TMs 3, 5, and 6, while the terminal imide was situated near TMs 1 and 2 and the extracellular side. These studies supported the hypothesis about the bioactive linear conformation of long-chain arylpiperazines as the ligands were consequently docked in a fully extended conformation.

**Figure 9:**
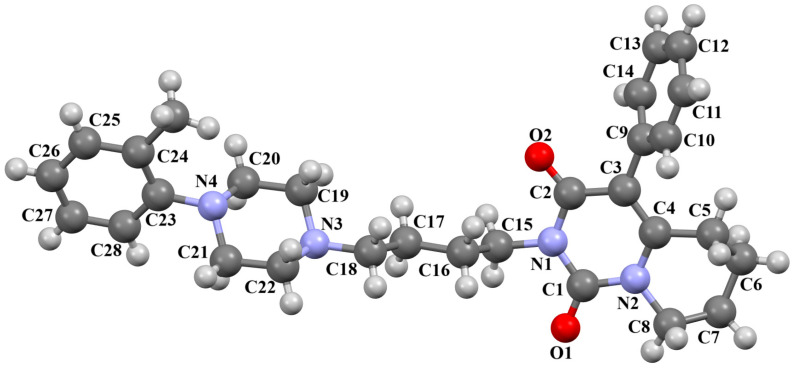
The molecular structure of LUYQOL [18,19,46].

Herold and co-workers reported the structure of arylpiperazine (LUYQOL, Figure 9) consisting of four units: an *o*-tolyl group, a piperazinyl moiety, an *n*-butyl linker, and a hexahydro-1*H*-pyrido[1,2-*c*]pyrimidine-1,3-dione fragment as the 5-HT_1A_ and 5-HT_2A_ receptor ligand [46]. The phenyl (C23/C24/C28/C27/C26/C25) and 1*H*-pyrido[1,2-*c*]pyrimidine (N1/C1/N2/C8/C7/C6/C5/C4/C3/C2) rings are inclined to piperazine by 65.07° and 16.78°, respectively. The −(CH_2_)_2_− linker adopts an *anti*−conformation (176.3(3)°). The same conformation is observed for the N3−C18−C17−C16 (−179.2(3)°) and C17−C16−C15−N1 (165.5(3)°) fragments.

**Figure 10:**
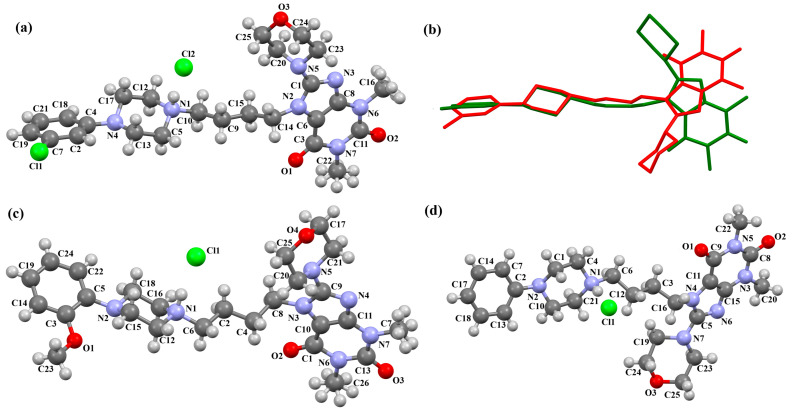
(**a**) The molecular structure of SOBVOW; (**b**) A comparison of the conformations of SOBVOW (green) and SOBVUC (red) with respect to the piperazine ring; (**c**) The molecular structure of SOBWAJ; (**d**) The molecular structure of SOBWEN [18,19,55]. The solvent molecules have been omitted.

The structures of four 7-arylpiperazinylalkyl-8-morpholin-4-yl-purine-2,6-dione derivatives (SOBVOW, SOBVUC, SOBWAJ, and SOBWEN) differing in the type and position of the substituent in the aryl ring as the 5-HT1_A_, 5-HT_2A_, 5-HT_6_, and 5-HT_7_ receptor ligands have been described by Chłoń-Rzepa et al. [55]. All compounds were obtained as hydrated hydrochloride salts. The compounds SOBVOW and SOBVUC contain a chlorine atom in the benzene ring in the *meta* position (Figure 10a) and differ in the number of solvent molecules (Table 3), which affects their structure. The first difference can be seen in the rotation of the plane of the phenyl ring relative to the plane of the piperazine ring. In the case of SOBVOW, the dihedral angle is 27.98°, whereas for SOBVUC, it is only 8.33°. Significant differences are also observed in the position of the plane of the tetrahydropurine ring in relation to the plane of the piperazine, i.e., in the compound SOBVOW, it is almost perpendicular with a dihedral angle of 87.10°, whereas in SOBVUC, a much lower angle value is observed (42.42°). A comparison of the SOBVOW and SOBVUC conformations is shown in Figure 10b. In both compounds, the *n*-butyl linker (−(CH_2_)_4_−) has an antiperiplanar conformation with angles of 173.0(2)° and 171.2(2)° for SOBVOW and SOBVUC, respectively. The next compound, SOBWAJ, contains a methoxy substituent (Figure 10c). The planes of phenyl (C5/C3/C14/C19/C24/C22) and tetrahydropurine (N3/C9/N4/C11/N7/C13/N6/C1/C10) rings are inclined to the piperazine ring plane (N2/C15/C12/N1/C16/C18) by 39.29° and 72.66°, respectively. The spacer −(CH_2_)_4_−, which has an antiperiplanar conformation (torsion angle of C6−C2−C4−C8 is −172.7(2)°), links the fused rings unit to the piperazinyl moiety in an *anti-anti* conformation with torsion angles of 167.5(2)° (N1−C6−C2−C4) and 166.5(2)° (C2−C4−C8−N3). The last derivative, SOBWEN, does not contain any substituents in the phenyl ring (Figure 10d). The arylpiperazine moiety in this structure also exhibits a non-coplanar conformation with the piperazine plane (formed by N2/C10/C21/N1/C4/C1 atoms) inclined to the phenyl plane (C2/C7/C14/C17/C18/C13) by 17.57°. The tetrahydropurine plane is also slightly twisted with respect to piperazine. The dihedral angle between the planes formed by the non-hydrogen atoms is only 15.86° and is the smallest compared to the other 7-arylpiperazinylalkyl-8-morpholin-4-yl-purine-2,6-dione derivatives mentioned. The tetramethyl linker adopts an *anti*−conformation with torsion angles of 175.6(2)° (N1−C6−C12−C3), 171.5(2)° (C6−C12−C3−C16), and 164.4(2)° (C12−C3−C16−N4).

**Figure 11:**
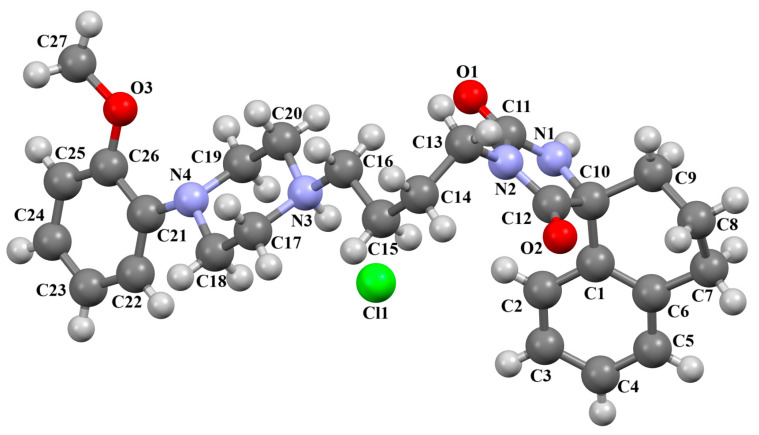
The molecular structure of VUFTIB [18,19,58].

The VUFTIB was synthesized as hydrochloride salt (Figure 11) with the positive charge localized on the N3 atom of the piperazine ring [58]. The compound is non-planar and the plane of the heterocyclic ring formed by the N2/C11/N1/C10/C12 atoms is approximately perpendicular to the plane of the piperazine ring (N3/C17/C18/N4/C19/C20), with the dihedral angle being 80.29°. Less rotation occurs within the arylpiperazine fragment, in which the angle between the phenyl (C21/C22/C23/C24/C25/C26) and piperazine planes is 41.84°. The −(CH_2_)_4_− linker (C13−C15−C16−C11) has a *gauche* conformation with a torsion angle of −58.9(2)°. This spacer links the heterocyclic fragment to the piperazinyl moiety in an *anti–gauche* conformation with torsion angles of −179.0(1)° (N3−C16−C15−C14) and −55.9(2)° (C15−C14−C13−N2).

The compound described above was flexibly docked to homology models of 5-HT_1A_ and D_2_ receptors. As the tested compound was synthesized in a racemic form, both the enantiomers were considered in molecular modeling studies. Due to better scores and a higher number of favorable interactions in both receptors, the S enantiomer was regarded as preferable and its binding mode was further described [58]. As expected for aminergic GPCRs, the key interaction was a charge-reinforced hydrogen bond between the protonatable nitrogen atom of the ligand and carboxyl group of Asp^3.32^. It was accompanied by the CH–π interactions of arylpiperazine and an aromatic aminoacid cluster, mainly Phe^6.52^. The conformation of the compound was linear in both receptors. The molecule extended from the deeper cavity formed by transmembrane helices 3–6 to the second interaction area, situated between TMs 1, 2, and 7.

#### 2.2.3. Arylpiperazine Derivatives Containing Flexible Aliphatic Chain with Heteroatom

**Figure 12:**
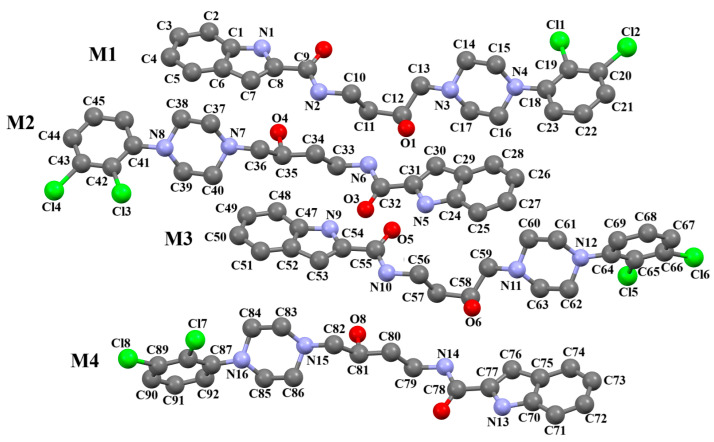
Molecular structure of KADXOE [18,19,44]. The disordered chlorine and phenyl ring atoms, hydrogen atoms, and solvent molecules have been omitted for clarity.

The structure of the enantioselective D_3_ receptor antagonist KADXOE, shown in Figure 12, was reported by Newman and co-workers [44]. In the asymmetric unit, there are four independent molecules. They are arranged in an alternating head-to-tail fashion. The compound is a 2,3-dichlorophenylpiperazine derivative containing a 1*H*-indole-2-carboxyamide moiety linked to the 2,3-dichlorophenyl ring via 3-hydroxybutane at the 1 and 4 positions. The phenyl ring plane is significantly inclined to the piperazine ring plane in all molecules. The interplanar angles are 61.13° (M1), 48.68° (M2), 45.86° (M3), and 47.87° (M4). A greater degree of rotation relative to piperazine is observed for the indole plane with the dihedral angels being 72.23° (M1), 59.88° (M2), 76.44° (M3), and 64.10° (M4), respectively. The tetramethylene fragment adopts *anti*, *gauche*, *anti* (for M1 and M3) or *anti*, *anti*, *anti*−conformations (for M2 and M4).

**Figure 13:**
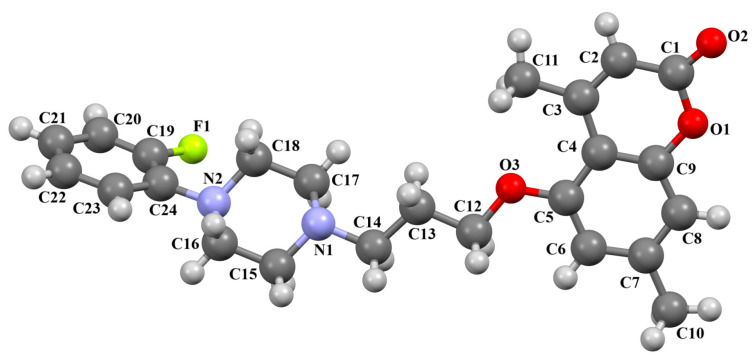
The molecular structure of KAXQEI [18,19,45].

Ostrowska et al. [45] synthesized an arylpiperazine derivative as the 5HT_1A_ receptor ligand consisting of the following units: a dichlorophenyl ring, a piperazinyl moiety, a propoxy linker, and a 4,7-dimethylcoumarin fragment (Figure 13). The planes of the phenyl ring (C24/C23/C22/C21/C20/C19) and the coumarin fragment (C5/C6/C7/C8/C9/O1/C1/C2/C3/C4) are inclined to the piperazine plane (N1/C17/C18/N2/C16/C15) by 41.73° and 43.02°, respectively. The propoxy linker is elongated and adopts an *anti*, *anti*, *anti*–conformation with torsion angles equal to 179.9(1)° (N1−C14−C13−C12), 175.3(1)° (C14−C13−C12−O3), and −177.1(1)° (C13−C12−O3−C5).

The compound described above was docked to the homology model of the 5-HT_1A_ receptor using AutoDock 4.2 [45]. Based on the computational results, an attempt was made to rationalize the obtained experimental *K_i_* values. The studied ligand forms multiple favorable interactions with the receptor binding pocket. The most important is the salt bridge between the protonatable piperazine nitrogen atom and Asp^3.32^. Furthermore, the hydrogen bonds are observed between Ser^5.43^ and the fluorine atom of the phenyl group and Tyr^7.42^ and the acetyl group. Next, multiple van der Waals interactions were found that stabilize both parts of the ligand. The studied compound displays a relatively high level of flexibility and can form relatively strong and favorable interactions, which translate into a very low *K_i_* value [45].

**Figure 14:**
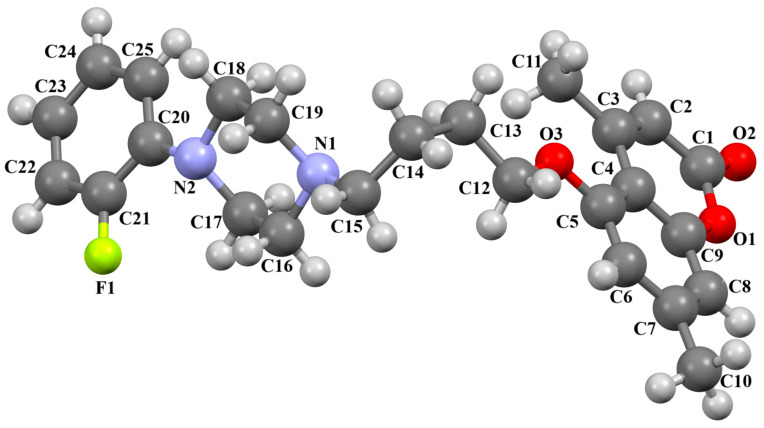
The molecular structure of ZOQPOM [18,19,60].

Ostrowska and co-workers also synthesized the compound ZOQPOM [60] containing the 4,7-dimethylcoumarin unit linked to the *o*-fluorophenylpiperazine fragment via the butylformamide linker (Figure 14). The planes of the phenyl ring (C20/C21/C22/C23/C24/C25) and the coumarin fragment (C5/C6/C7/C8/C9/O1/C1/C2/C3/C4) are rotated to the piperazine plane (N1/C19/C18/N2/C17/C16) by 37.59° and 69.22°, respectively. The −(CH_2_)_4_− fragment (C15–C14–C13–C12) has a *gauche* conformation; the torsion angle is −73.5(1)°. The *n*-butoxyl fragment adopts an *anti–gauche* conformation, causing the molecule to be bent. The torsion angles of O3–C12–C13–C14 and C13–C13–C14–N1 are 173.6(1)° and −57.8(1)°, respectively.

**Figure 15:**
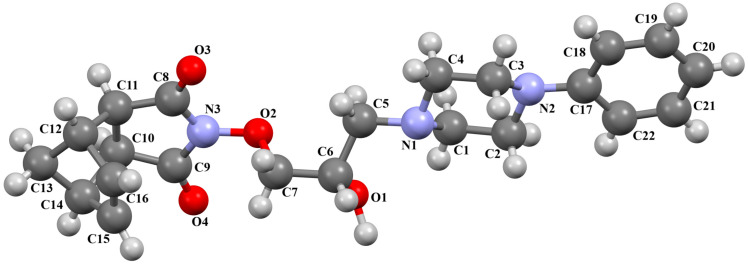
The molecular structure of POPDUU [18,19,48].

In the next compound, POPDUU (Figure 15), the phenyl ring is slightly rotated with respect to the piperazine fragment, and the dihedral angle between the planes formed by non-hydrogen atoms is 13.0°. The 2-hydroxypropyl linker is bent, and the C5−C6−C7−O2 fragment has a *gauche* conformation with torsion angles of 79.2(4)°. It binds the piperazine ring and 4-azatricyclo[5.2.1.0^2,6^]dec-8-ene-3,5-dione unit in an *anti–anti* conformation with torsion angles of 166.3(3)° (N1−C5−C6−C7) and −175.3(1)° (C6−C7−O2−N3) [48].

**Figure 16:**
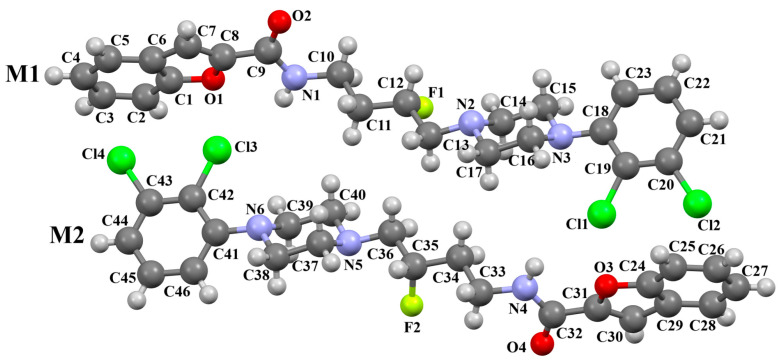
The molecular structure of QOQYAY [18,19,54].

The compound QOQYAY, a selective dopamine D_3_ receptor antagonist synthesized by Kumar et al., consists of a coumarone unit linked to the 2,3-dichlorophenylpiperazine fragment via a butylformamide linker [54]. In the asymmetric unit, there are two, non-planar independent molecules (Figure 16). The planes of the phenyl and piperazine rings are rotated against one another by angles of 48.47° for M1 and 48.87° for M2. In the case of the coumarone plane, it is inclined to the piperazine plane by 52.73° for M1 and 52.64° for M2. In both molecules, the tetramethylene fragment adopts an antiperiplanar conformation.

**Figure 17:**
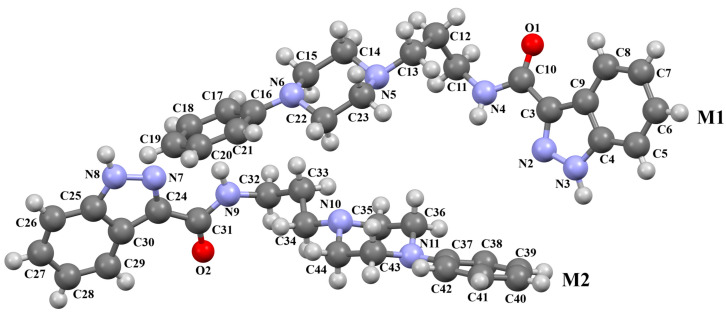
The molecular structure of TOYTIO [18,19,56]. The solvent molecule has been omitted.

The compound TOYTIO, a multi-target ligand of aminergic GPCRs, obtained by Stępnicki and co-workers, crystallized as an acetylonitrile solvate containing two independent molecules (M1 and M2) in an asymmetric unit arranged head-to-tail (Figure 17) [56]. The molecules M1 and M2 are non-planar. The dihedral angles between the planes formed by the non-hydrogen atoms of the piperazine ring and indazole fragment are 24.51° and 19.35° for M1 and M2, respectively. The phenyl ring plane is more rotated with respect to piperazine, the dihedral angles are 47.50° (M1) and 82.88° (M2). The molecules also differ in the conformation of the linker −(CH_2_)_3_NHC(O)−). In the molecule M1, the spacer adopts *gauche*, *gauche*, *gauche*, *anti*−conformations with torsion angles of −67.7(4)° (N5−C13−C12−C11), −56.2(4)° (C13−C12−C11−N4), 77.0(4)° (C12−C11−N4−C10), and −173.3(3)° (C13−N4−C13−C12), while in M2, *anti*, *gauche*, *eclipsed*, *anti*−conformations are observed with torsion angles of 174.8(3)° (N10−C34−C33−C32), 67.7(4)° (C34−C33−C32−N9), −133.0(4)° (C33−C32−N9−C31), and −171.7(3)° (C32−N9−C31−C24), respectively.

The studied compound was docked with Glide to the binding pockets of the D_2_ and 5-HT_2A_ receptors in the inactive state (X-ray structures) and the 5HT_1A_ receptor in the active state (cryo-EM structure). The compound shows a very similar arrangement in the binding sites of the investigated receptors [56]. As expected, the main contact is the salt bridge between the protonatable nitrogen atom of the ligand and Asp^3.32^. In the case of the D_2_ receptor, there are π–π stacking interactions between the phenyl group attached to the piperazine and the aromatic systems of Phe^6.52^ and Trp^6.48^. Next, the hydroxyl group of Tyr^7.43^ forms a hydrogen bond with the carbonyl group of the ligands. In the 5-HT_1A_ receptor, there are π–π stacking interactions of the phenyl group of the ligand with Phe^6.52^ and between the indazole fragment and Tyr^2.64^. In the 5-HT_2A_ receptor, there are also π–π stacking interactions between an aryl substituent at the piperazine moiety and the side chains of the aromatic amino acids Phe^6.52^ and Trp^6.48^.

**Figure 18:**
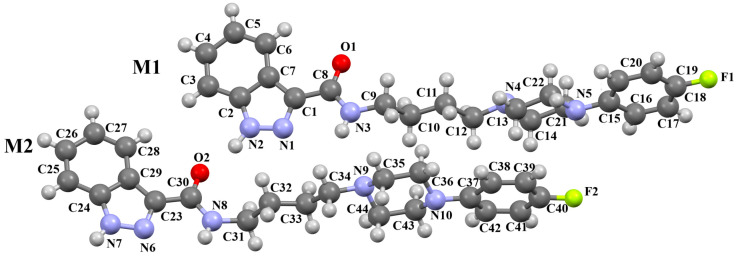
The molecular structure of ZIGWOF [19,46,59].

Stępnicki and co-workers also synthesized an arylpiperazine derivative (ZIGWOF) with a longer spacer, i.e., a butylformamide linker as a multi-target ligand of aminergic GPCRs [59]. As in the previous compound, there are two molecules in an asymmetric unit, but in a head-to-head arrangement (Figure 18). The phenylpiperazine fragment is not planar. The plane of the phenyl ring, formed by non-hydrogen atoms, is rotated relative to the piperazine ring by 25.31° for M1 and 10.68° for M2. The planes of the piperazine ring and the indazole scaffold are more inclined towards each other, and the dihedral angles are 82.88° for M1 and 79.20° for M2, respectively. The tetramethylene fragment adopts an antiperiplanar conformation in both molecules.

The studied compound was docked to the experimental structures of the D_2_, 5-HT_1A_ and 5-HT_2A_ receptors as described above. The pattern of interactions with the receptor binding pockets is also similar [56,59].

#### 2.2.4. Arylpiperazine Derivatives Containing Flexible Aliphatic Chain with an Electron-Donating Substituent

**Figure 19:**
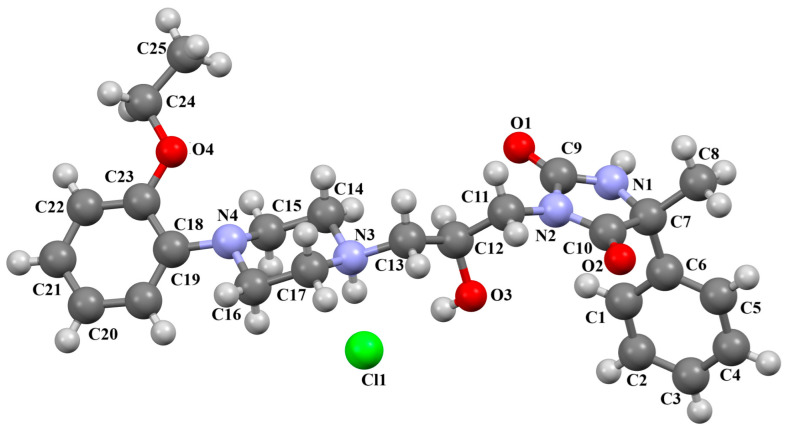
Molecular structure of DEMSAS [18,19,42]. The solvent molecule (*n*-decane) has been omitted.

The DEMSAS compound, a serotonin 5-HT_7_ receptor ligand, was synthesized as a hydrochloride salt solvate (Figure 19) [42]. The molecule is non-planar. The dihedral C1/C2/C3/C4/C5/C6 is very nearly perpendicular to the plane of the heterocyclic ring (86.72°). The hydantoin ring is connected to the piperazinyl unit via the >(CH_2_)_2_CHOH linker. The conformation of the aliphatic chain is *anti–anti*, with the torsion angles of N3−C13−C12−C11 and C11−C12−C13−N12 being 171.3(1)° and 179.4(1)°, respectively.

To study the ligand–receptor interactions at the molecular level, molecular docking and molecular dynamics simulations were performed [42]. The main ligand–receptor interactions involved the following: a salt bridge with Asp^3.32^, CHπ/π–π interactions between the 2-ethoxyphenylpiperazine moiety and the side chains of Phe^6.51^ and Phe^6.52^, and π–π stacking interactions with Phe^3.28^ and the hydrogen bond between the hydantoin fragment and Cys146 of extracellular loop 2 (ECL2). The MD simulations additionally indicated that the hydantoin moiety is stabilized by hydrogen bonds with Arg^7.36^ and Arg^6.58^. The compound stayed in the 5-HT7 receptor binding cavity for the entire simulations and adopted a conformation close to those obtained from the docking and X-ray studies.

**Figure 20:**
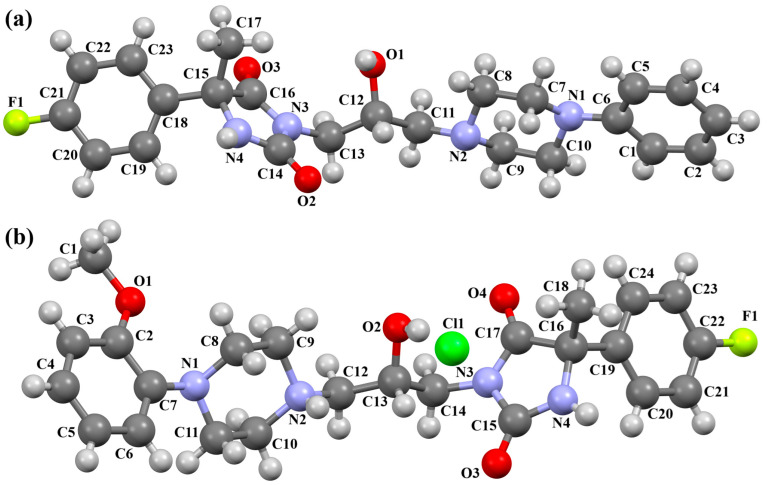
(**a**) The molecular structure of QEYBAA [18,19,52]. (**b**)The molecular structure of WAGMOK [18,19,53]. The solvent molecule (isobutanol) has been omitted.

Kucwaj-Brysz et al. synthesized arylpiperazine derivatives with the 2-hydroxypropyl spacer QEYBAA and WAGMOK (Figure 20) [52,53]. WAGMOK crystallized as a solvated hydrochloride salt. In the case of QEYBAA, the phenyl ring is roughly coplanar with the piperazine ring (N1/C7/C8/N2/C9/C10) with a dihedral angle of 7.45°, while for WAGMOK, it is inclined by 45.28°. The flexible hydroxytrimethylene linker, which binds hydantoin and piperazine rings, shows an extended *anti–anti* conformation, with the torsion angles of −179.4(2)° (N3−C12−C13−C11) and −147.8(2)° (C13−C12−C11−N2) for QEYBAA and of 168.3(1)° (N2−C8−C7−C6) and −176.0(1)° (C8−C7−C3−N3) for WAGMOK.

The investigated compounds were docked to a homology model of the 5-HT_7_ receptor. Following the pharmacophore model for the 5-HT_7_ receptor ligands, they formed a salt bridge with Asp^3.32^ and interacted with the aromatic cluster formed by Phe6.51 and Phe6.52. The lack of 5-HT7 receptor affinity in some derivatives of QEYBAA has been attributed to the lack of electrostatic interactions with Asp^3.32^ [52]. In the case of WAGMOK derivatives, some interactions with the residues of ECL 2 (Cys162, Leu163, and Ile164) were also found [53]. In general, WAGMOK, as the most active compound of the series, adopted a significantly different pose compared to the other stereoisomers tested, located deepest in the binding pocket. The stability of this ligand–receptor complex was confirmed in molecular dynamics simulations.

**Figure 21:**
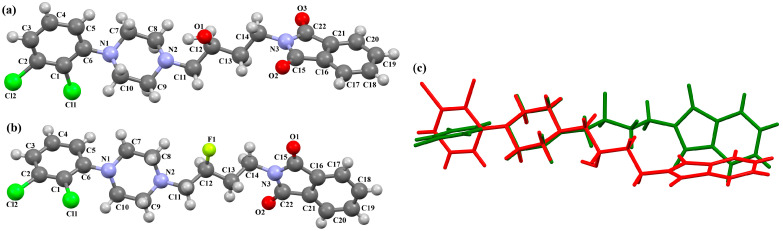
The molecular structure of (**a**) QOQXOL; (**b**) QOQXUR [18,19,54]. (**c**) A comparison of the conformations of QOQXOL (red) and QOQXUR (green).

The compounds QOQXOL and QOQXUR (Figure 21), D_3_ receptor antagonists, synthesized by Kumar et al. [54], differ in the linker, i.e., the electron-donating substituent in the butyl chain (OH group or fluorine). In both compounds, the plane of the phenyl ring is rotated with respect to the piperazine plane by 55.32° for QOQXOL and 49.93° for QOQXUR, respectively. A smaller degree of rotation is observed for the isoindoline plane in relation to the piperazine ring plane. The interplanar angles are 32.75° (QOQXOL) and 36.97° (QOQXUR). The hydroxytetramethylene linkers show an extended conformation, with the torsion angles being 165.2(2)° (N2–C11–C12–C13), −175.2(2)° (C11−C12−C13−C14), and −178.7(2)° (C12−C13−C14−N3) for QOQXOL and −150.6(2)° (N2−C11−C12−C13), −174.9(2)° (C11−C12−C13−C14), and 174.1(2)° (C12−C13−C14−N3) for QOQXUR, respectively. A comparison of the QOQXOL and QOQXUR conformations is shown in Figure 21c.

**Figure 22:**
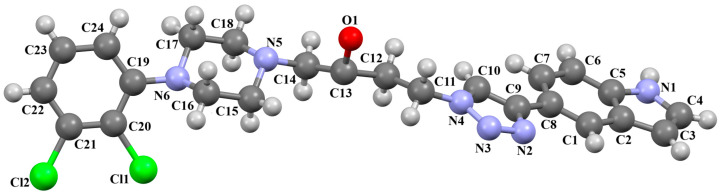
The molecular structure of VOTDUF [18,19,57].

A D_3_ receptor ligand, VOTDUF, is reported as a 2,3-dichlorophenylpiperazine derivative containing an indolyltriazole fragment linked to the arylpiperazine fragment via 2-hydroxybutane (Figure 22) [57]. The plane of the phenyl ring (C19/C20/C21/C22/C23/C24) is significantly inclined to the plane of the piperazine ring (N6/C17/C18/N5/C15/C16) by 41.84°. A smaller interplanar angle of 25.17° is observed for triazole (N4/N3/N2/C9/C10) and piperazine rings. The tetramethylene fragment adopts an *anti*, *anti*, *anti*−conformation with torsion angles of −177.1(6)° (N5−C14−C13−C12), −168.2(6)° (C14−C13−C12−C11), and 172.0(4)° (C13−C12−C11−N4), respectively.

#### 2.2.5. Other Arylpiperazine Derivatives

**Figure 23:**
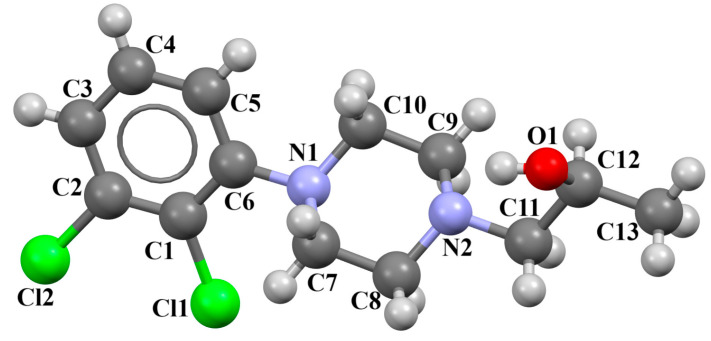
The structure of BAHRIO [18,19,39].

Michino et al. obtained two structures of the arylpiperazine derivative, i.e., (*R*)- and (*S*)-1-(4-(2,3-dichlorophenyl)piperazin-1-yl)propan-2-ol as ligands of the D_3_ receptor [39]. The structure of the (*S*)-enantiomer is shown in Figure 23. In both compounds, the interplanar angles between the piperazine and phenyl rings (the plane formed by the non-hydrogen atoms) are similar and equal 43.40° and 43.48° for the (*S*)- and (*R*)-enantiomers, respectively. The torsion angles *τ_1_* (C13−C13−C11−N2) and *τ_2_* (O1−C13−C11−N2) are 176.6(1) and 57.5(2) for the (*S*)-enantiomer and −176.8(2) and −57.6(2) for the (*R*)-enantiomer.

**Figure 24:**
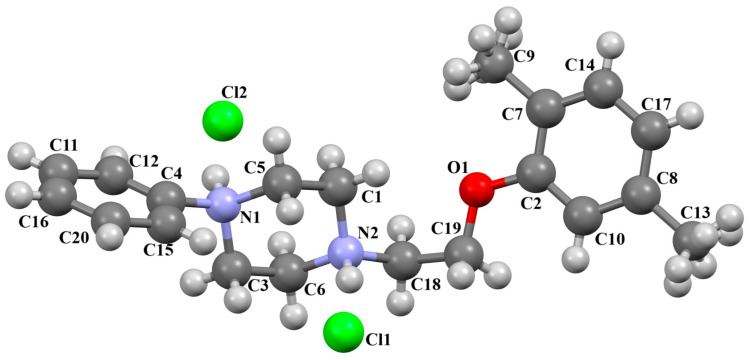
The molecular structure of POVSAX [18,19,49].

Pańczyk and co-workers synthesized an arylpiperazine derivative in the form of a dihydrochloride salt (Figure 24) with the positive charges localized on both nitrogen atoms of the piperazine ring, as CNS agents [49]. The compound is non-planar. Both phenyl ring planes are inclined to the piperazine plane (N1/C3/C6/N2/C1/C5) by 75.82° (C4/C15/C20/C16/C11/C12) and 45.89° (C2/C10/C8/C17/C14/C7). The ethoxy linker adopts a *sc–ap* conformation with torsion angles of −80.8(4)° (N2−C18−C19−O1) and −169.1(3)° (C18−C19−O1−C2).

Flexible docking studies of the above compound were performed for homology models of the 5-HT_1A_, 5-HT_2A_, and 5-HT_7_ receptors. The compound displayed an extended conformation and was located across the two parts of the binding site: the deeper one formed between TMs 3–6 and the second one, located between TMs 2 and 7 [49]. The main interactions between POVSAX and the studied serotonin receptors included a charge-reinforced hydrogen bond between the protonatable nitrogen atom of phenylpiperazine and the carboxyl group of Asp^3.32^, as well as the CH-π stacking with Phe^6.52^.

**Figure 25:**
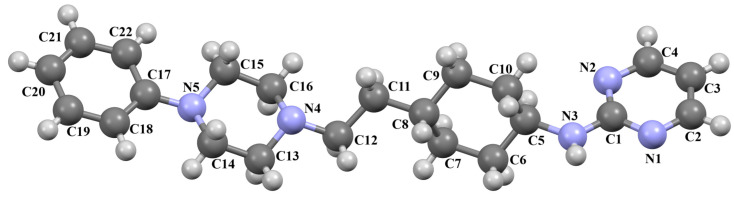
The molecular structure of PUDGOK [18,19,50].

The PUDGOK is an arylpiperazine derivative containing a pyrimidine ring linked to a phenylpiperazine fragment via the 4-ethylcyclohexanamine moiety (Figure 25) and exhibits a high affinity to serotonin and dopamine receptors [50]. The phenyl ring (C17/C18/C19/C20/C21/C22) is almost coplanar with the piperazine ring (N5/C15/C16/N4/C13/C14) with an interplane angle of 5.68°. A slightly larger rotation with respect to the piperazine plane is observed for the pyrimidine ring (C1/N1/C2/C3/C4/N2), for which the dihedral angle is 14.88°. The cyclohexane ring, like the piperazine ring, adopts a chair conformation.

**Figure 26:**
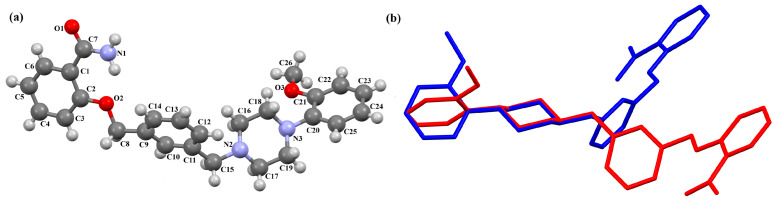
(**a**) Molecular structure of QENURA [18,19,51]; (**b**) Comparison of methanol and ethanol solvates of 2-[(3-{[4-(2-methoxyphenyl)piperazin-1-yl]methyl}phenyl)methoxy]benzamide (QENURA—blue, QENSOV—red). The solvent molecules have been omitted.

Pindelska and co-workers synthesized 2-[(3-{[4-(2-methoxyphenyl)piperazin-1-yl]methyl}phenyl)methoxy]benzamide (Figure 26a) in the form of two different solvates (methanol—QENURA or ethanol—QENSOV) [51]. The compound is an arylpiperazine derivative that does not contain a heterocyclic fragment but salicylamide, which is attached via a dimethylbenzene linker to the phenylpiperazine. The structures of the compounds are not planar. A significant rotation of the phenyl ring (C20/C21/C22/C23/C24/C25) with respect to the piperazine ring (N2/C16/C18/N3/C19/C17) is observed in both of the solvates. The dihedral angles between these planes are 47.47° and 48.74° for the methanol and ethanol solvates, respectively. The plane (C9/C10/C11/C12/C13/C14/C15) of the dimethylbenzene linker (−CH_2_(C_6_H_4_)CH_2_−) is more twisted relative to the piperazine ring. The interplanar angles are 71.65° (QENURA) and 70.11° (QENSOV). A comparison of the methanol and ethanol solvates is shown in Figure 26b.

## 3. Conclusions

Arylpiperazines are among the most important classes of aminergic GPCR ligands. This scaffold is present in many CNS drugs, including trazodone and aripiprazole. This review compiles structural information on the solid-state conformations of arylpiperazines derived from X-ray studies. There are some examples of compounds which crystallize as hydrochloride salts with the positive charge mainly located on the nitrogen atom attached to the linker. In all the structures of the presented arylpiperazines, the piperazine ring adopts a chair conformation with substituents in an equatorial position. The aryl moiety is generally significantly rotated with respect to the piperazine ring, similar to the heterocyclic moiety. In most cases, the linker is found in the *anti*-conformation, which causes the compounds to have an elongated form. This was supplemented by a discussion of ligand–receptor interactions based on experimental or modeled ligand–receptor complexes when available. The described arylpiperazines displayed a similar pattern of interactions with the aminergic GPCRs with Asp^3.32^ as the major anchoring residue for a protonatable nitrogen atom of the piperazine moiety. Furthermore, Phe^6.51^ and Phe^6.52^ were often found to interact with the ligands.

Low-energy conformations of the molecules found in the solid state are presented, along with only two experimentally confirmed bioactive conformations. As stated in the introduction, these conformations are different in principle. Furthermore, only a few molecular dynamics simulations are described, providing additional information about the flexibility of these molecules. These are the main limitations of our review. However, our collection of molecular structures of *N*-arylpiperazines illustrates their diversity and provides a straightforward suggestion for the use of ligand- or structure-based design.

## Figures and Tables

**Table 1:** Geometrical details of polymorphs of aripiprazole [19].

CSD Refcode	Space Group	∠A/B (°)	∠C/B (°)	*τ_1_* (°)	*τ_2_* (°)	*τ_3_* (°)	*τ_4_* (°)	Ref.
MELFIT	*P*-1	M1: 45.47 M2: 48.18	M1: 52.22 M2:59.70	M1: −163.7(2) M2: 178.2(2)	M1: −178.9(2) M2: −175.5(2)	M1: −177.5(2) M2: 161.9(2)	M1: 172.4(2) M2: −174.7(2)	[20]
MELFIT01	*P*2_1_	44.90	52.61	173.7(6)	63.0(9)	−174.7(6)	−60.2(7)	[21]
MELFIT02	*Pna*2_1_	44.80	86.20	−176.2(9)	178.9(9)	173.3(9)	−178.0(9)	[21]
MELFIT03	*P*-1	M1: 46.81 M2: 49.86	M1: 52.80 M2: 59.71	M1: 166.1(7) M2: 179.7(8)	M1: 178.9(6) M2: −177.5(8)	M1: 175.9(7) M2: 161.8(9)	M1: −173.0(6) M2: −174.2(8)	[21]
MELFIT04	*P*-1	M1: 43.83 M2: 42.14	M1: 46.88 M2: 41.28	M1: 176.7(2) M2: −175.3(3)	M1: 179.6(3) M2: 175.2(3)	M1: 176.5(3) M2: 178.3(3)	M1: −169.4(3) M2: −179.6(3)	[21]
MELFIT05	*P*2_1_	41.03	41.96	178.8(3)	174.4(3)	169.6(3)	170.6(3)	[21]
MELFIT06	*P*-1	M1: 35.23 M2: 41.85	M1: 33.18 M2: 41.32	M1: 178.5(2) M2: −170.6(2)	M1: 178.6(2) M2: −178.5(2)	M1: −175.9(2) M2: −178.2(2)	M1: 168.9(2) M2: −171.0(2)	[22]
MELFIT07	*P*-1	45.48	81.35	−175.8(1)	−176.4(1)	−58.3(2)	−53.8(2)	[23]
MELFIT08	*Pna*2_1_	44.15	86.06	176.3(2)	−178.4(3)	−171.6(3)	179.0(2)	[24]
MELFIT09	*Pna*2_1_	47.86	87.61	169.2(1)	68.5(2)	179.8(2)	−56.0(2)	[24]
MELFIT10	*Pna*2_1_	47.78	87.64	−169.5(3)	−68.2(4)	−180.0(3)	56.5(4)	[24]
MELFIT11	*Pna*2_1_	47.80	87.68	−169.7(2)	−68.2(3)	−179.8(2)	55.9(3)	[24]
MELFIT12	*Pna*2_1_	47.87	87.70	169.9(3)	68.0(4)	179.8(3)	−56.2(4)	[24]
MELFIT13	*Pna*2_1_	47.99	87.87	170.2(2)	67.9(3)	179.6(3)	−56.4(3)	[24]
MELFIT14	*Pna*2_1_	47.69	87.64	−170.0(3)	−67.6(3)	−179.5(2)	56.8(3)	[24]
MELFIT15	*Pna*2_1_	44.24	86.16	176.0(2)	−178.3(2)	−171.2(2)	178.9(2)	[24]
MELFIT16	*Pna*2_1_	44.14	86.15	175.9(2)	−178.4(2)	−171.1(2)	179.1(2)	[24]
MELFIT17	*Pna*2_1_	44.30	86.16	176.0(2)	−178.8(2)	−171.5(2)	179.1(2)	[24]
MELFIT18	*Pna*2_1_	47.71	87.63	−168.9(2)	−68.1(3)	179.6(2)	53.8(3)	[24]
MELFIT19	*P*2_1_/*n*	53.11	82.61	179.4(1)	−177.5(1)	177.9(2)	55.3(2)	[25]
MELFIT20	*P*-1	M1: 40.16 M2: 42.78	M1: 40.46 M2: 45.41	M1: 175.1(2) M2: 177.8(2)	M1: −175.6(2) M2: −179.6(2)	M1: −177.9(2) M2: 176.9(2)	M1: −179.6(2) M2: −168.5(2)	[26]

M1—molecule 1, M2—molecule 2; A—plane formed by the non-hydrogen atoms of phenyl ring; B—plane formed by the non-hydrogen atoms of piperazine ring; C—plane formed by the non-hydrogen atoms of the fused ring (dihydroquinoline ring); A/B—dihedral angle between planes A and B; C/B—dihedral angle between planes C and B.

**Table 2:** The affinity of aripiprazole to selected dopamine and serotonin receptors [27].

Receptor	*K_i_* [nM]
D_1_	1960 ± 670
D_2L_	0.74 ± 0.09
D_2_	3.3 ± 1.1
D_3_	9.7 ± 5.4
D_3_	1.0 ± 0.40
rD_4_	510 ± 93
D_5_	2590 ± 1350
5-HT_1A_	5.6 ± 0.8
5-HT_1B_	830 ± 260
5-HT_1D_	68 ± 11
5-HT_1E_	8000 ± 5000
5-HT_2A_	8.7 ± 2.0
5-HT_2A_	35 ± 4
5-HT_2B_	0.36 ± 0.11
5-HT_2C_	76 ± 8
5-HT_5A_	1240 ± 280
5-HT_6_	570 ± 95
5-HT_7_	10.3 ± 3.7

**Table 3:** Scheme of arylpiperazine derivatives with CSD refcodes [18,19].

Scheme of the Compound	CSD Refcode	∠A/B (°)	Ref.
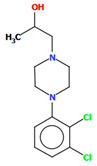	BAHRIO	43.40	[39]
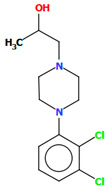	BAHROU	43.48	[39]
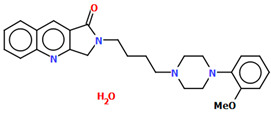	BIJBON	47.39	[40]
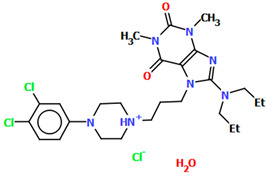	CAHLAB	27.26	[41]
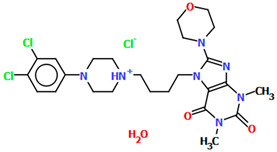	CAHLEF	5.39	[41]
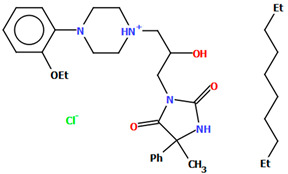	DEMSAS	43.76	[42]
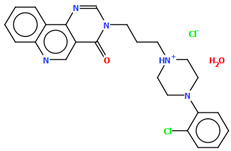	EZEYUE	53.69	[43]
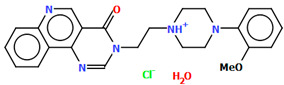	EZEZAL	49.03	[43]
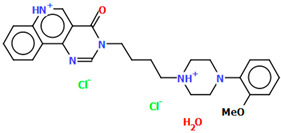	EZEZEP	45.75	[43]
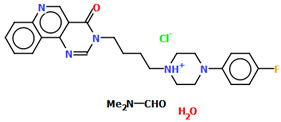	EZEZIT	3.77	[43]
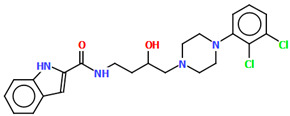	KADXOE	M1: 61.13 M2: 48.68 M3: 45.86 M4: 47.87	[44]
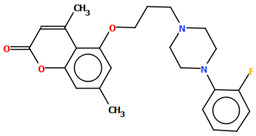	KAXQEI	41.73	[45]
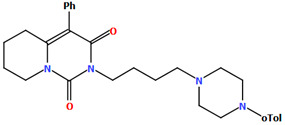	LUYQOL	65.07	[46]
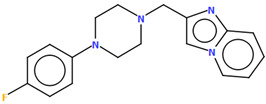	MIDVOL	34.56	[47]
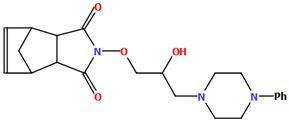	POPDUU	13.00	[48]
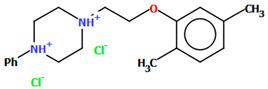	POVSAX	75.82	[49]
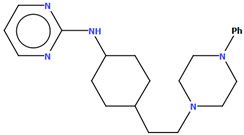	PUDGOK	5.68	[50]
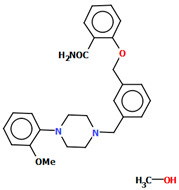	QENRUA QENRUA01 QENRUA02 QENRUA03	47.74 47.72 46.62 46.61	[51]
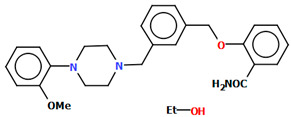	QENSOV QENSOV01	48.74 48.73	[51]
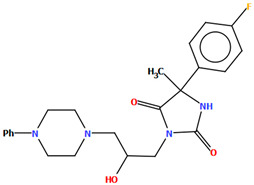	QEYBAA QEYBAA01	4.75 -	[52] [53]
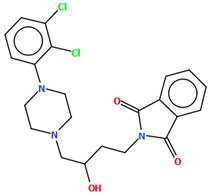	QOQXOL	55.32	[54]
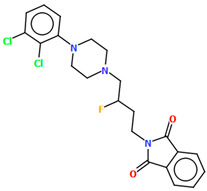	QOQXUR	49.93	[54]
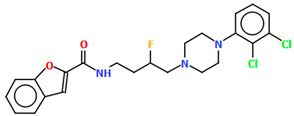	QOQYAY	M1: 48.47 M2: 48.87	[54]
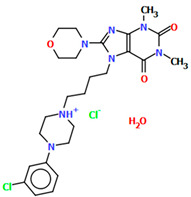	SOBVOW	27.98	[55]
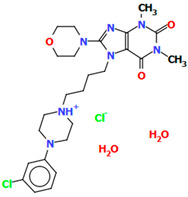	SOBVUC	8.33	[55]
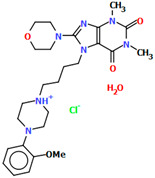	SOBWAJ	39.29	[55]
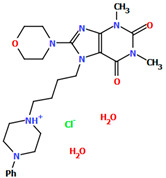	SOBWEN	17.57	[55]
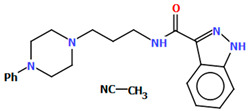	TOYTIO	M1: 24.51 M2: 19.35	[56]
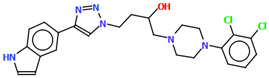	VOTDUF	44.33	[57]
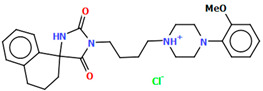	VUFTIB	41.84	[58]
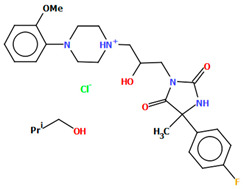	WAGMOK	45.28	[52]
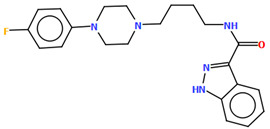	ZIGWOF	M1: 25.13 M2: 10.68	[59]
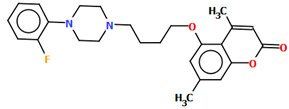	ZOQPOM	37.59	[60]

M1—molecule 1, M2—molecule 2, M3—molecule 3, M4—molecule 4; A—plane formed by the non-hydrogen atoms of phenyl ring; B—plane formed by the non-hydrogen atoms of the piperazine ring.

## Data Availability

No new data were created or analyzed in this study. Data sharing is not applicable to this article.

## Abbreviations

The following abbreviations are used in this manuscript:

CNS	Central nervous system
Cryo-EM	Cryogenic electron microscopy
ECL	Extracellular loop
ESR1	Estrogen receptor 1
GPCR	G protein-coupled receptors
MAPK3	Mitogen-activated protein kinase 3
LCAPs	Long chain arylpiperazines
PD	Parkinson’s disease
PPARG	Peroxisome proliferator-activated receptor gamma
TGA	Third-generation antipsychotics
TM	Transmembrane
